# Supplementary material for: CDK12 loss in cancer cells affects DNA damage response genes through premature cleavage and polyadenylation
Source: Nat Commun. 2019 Apr 15;10:1757. doi: 10.1038/s41467-019-09703-y (PMC6465371; doi:10.1038/s41467-019-09703-y)
Supplement: Supplementary file 1 — Supplementary Information [file 41467_2019_9703_MOESM1_ESM.pdf]

Supplementary Data File

CDK12 loss in cancer cells affects DNA damage response genes through premature cleavage and polyadenylation

Krajewska et al.

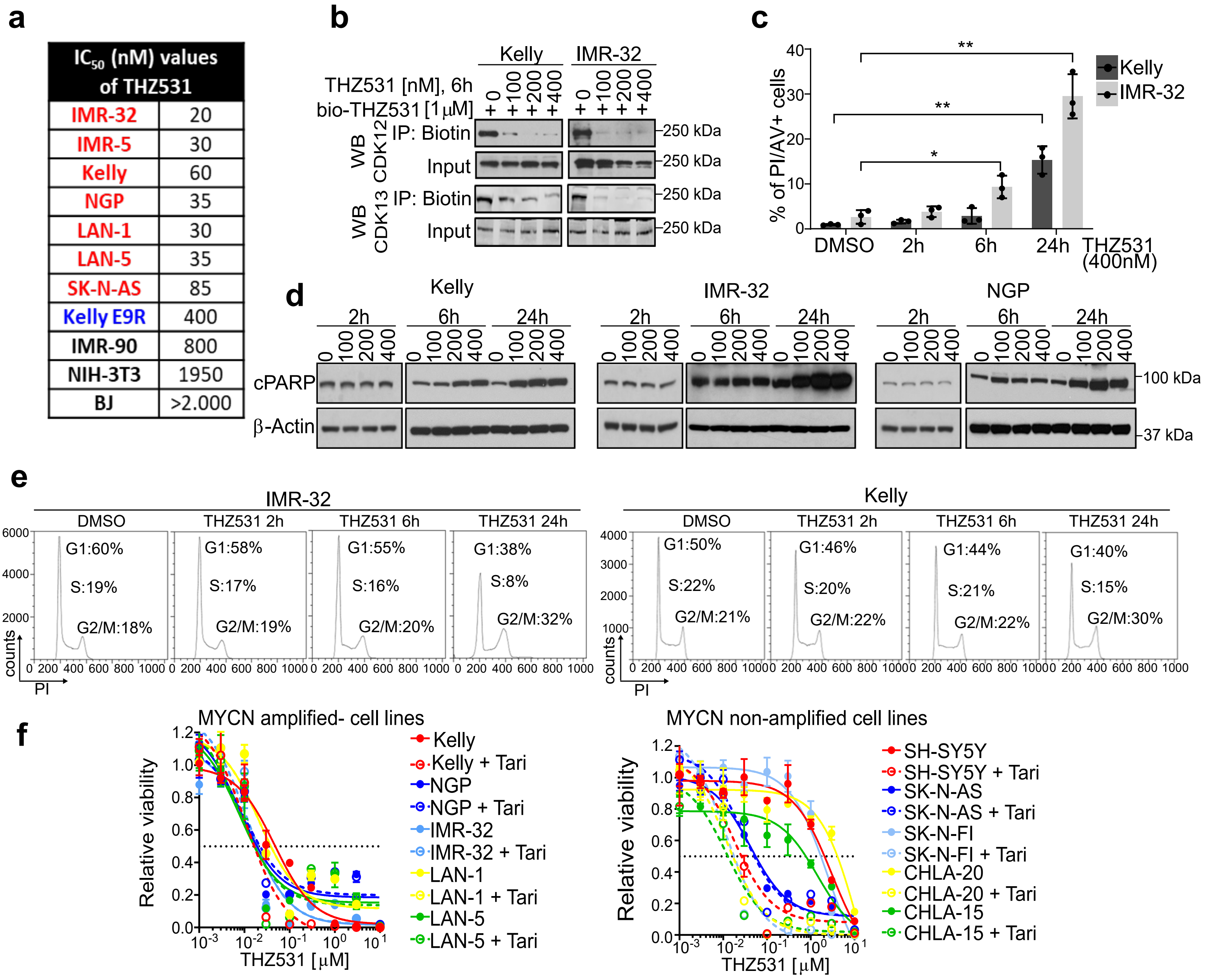

IC_{50} (nM) values of THZ531	
IMR-32	20
IMR-5	30
Kelly	60
NGP	35
LAN-1	30
LAN-5	35
SK-N-AS	85
Kelly E9R	400
IMR-90	800
NIH-3T3	1950
BJ	>2.000

IC_{50} (nM)		
Cell line	THZ531	
	-	+Tariq
Kelly	60	15
NGP	30	29
IMR-32	20	18
LAN-1	30	29
LAN-5	35	15

IC_{50} (nM)		
Cell line	THZ531	
	-	+Tariq
SK-N-AS	85	60
SH-SY5Y	2000	30
SK-N-FI	2100	43
CHLA-15	600	15
CHLA-20	3500	20

Supplementary Figure 1. CDK12/13 inhibition impairs viability of NB cells. a, IC_{50} values of THZ531 in NB vs. fibroblast (NIH-3T3, IMR-90, BJ) cells at 72 h. **b**, Analysis of target engagement in NB cells following THZ531 treatment. Cells were treated with THZ531 or DMSO for 6 h at the indicated concentrations and cell lysates incubated with 1 μM of biotinylated THZ531 (bio-THZ531) overnight. **c**, Flow cytometric analysis of Annexin V (AV) staining in Kelly and IMR-32 NB cells treated with 400 nM THZ531 for the indicated times. Error bars indicate mean values ± SD, n=3, *p<0.05, **p<0.01, two-tailed Student's t-test. **d**, Western blot analysis of cleaved PARP in Kelly, IMR-32 and NGP cells following treatment with THZ531 at the indicated doses and times. **e**, Cell-cycle analysis of NB cells exposed to THZ531 (400 nM for 2, 6 and 24 h), by flow cytometry with propidium iodide (PI) staining. Results are representative of three replicate experiments. **f**, Dose-response curves for MYCN-amplified (left) and non-amplified (right) human NB cells. Cells were treated with increasing concentrations of THZ531 alone or in combination with the ABCB1 inhibitor, tariquidar (125 nM) for 72 h. Percent cell viability relative to DMSO-treated cells is shown. Data represent mean ± SD; n=3.

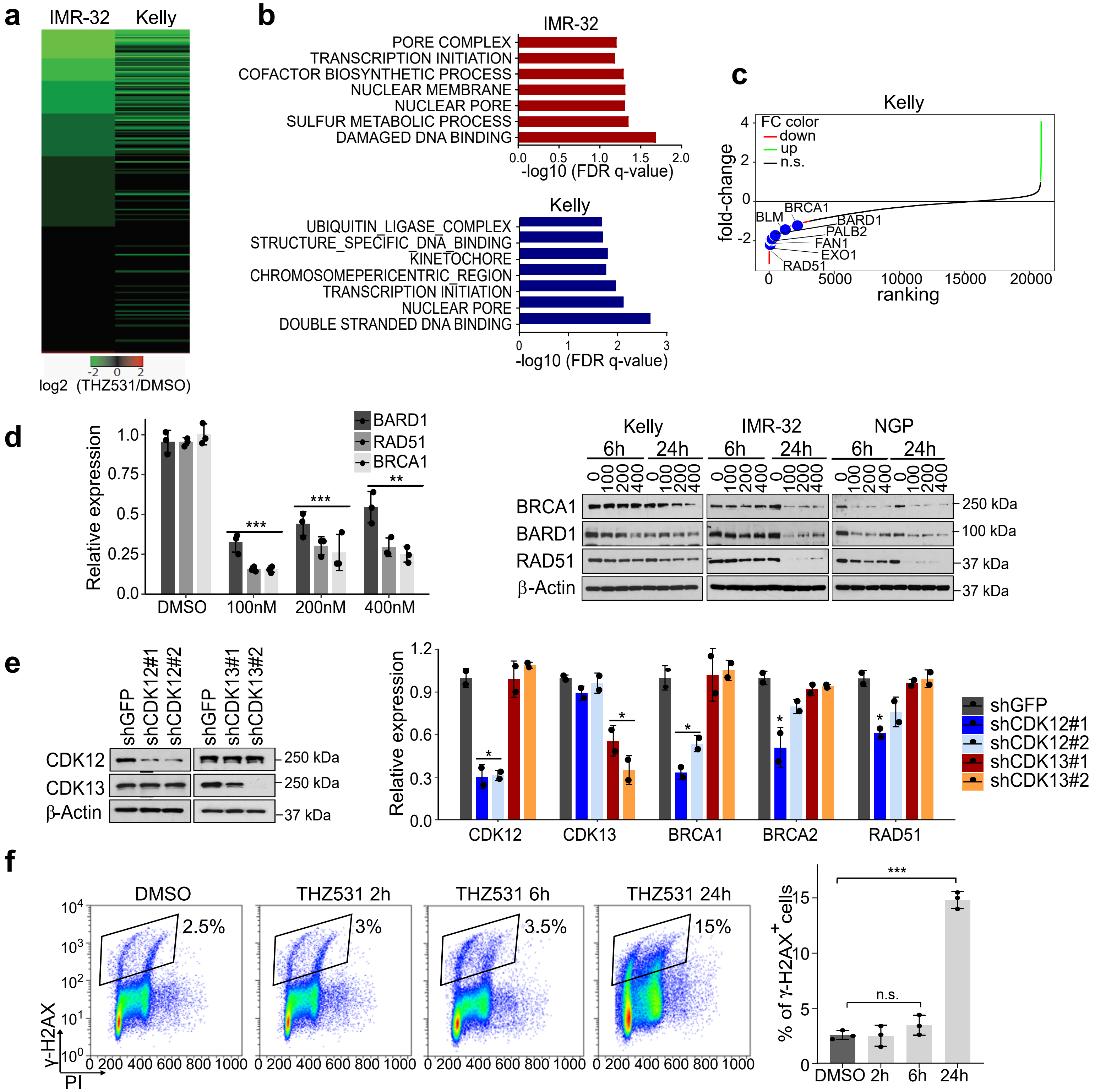

Supplementary Figure 2. CDK12/13 inhibition with THZ531 preferentially affects DDR genes. a, Heat map of gene expression values in NB cells treated with THZ531 (400 nM for 6 h) vs. DMSO. **b**, Gene set enrichment analysis (GSEA) of downregulated genes in NB cells treated as in panel a. **c**, Waterfall plot of log2 fold-changes in gene expression in Kelly NB cells treated as in panel a; selected DDR genes are highlighted. **d**, qRT-PCR (left) and immunoblot (right) analyses of selected DDR gene expression in NB cells treated with THZ531 at the indicated concentrations for 6 h. **e**, Immunoblot analysis (left) of CDK12 and CDK13 expression following shRNA knockdown in NB cells. β-actin was used as a loading control. qRT-PCR (right) of selected DDR expression in NB cells expressing either a control shRNA or two individual shRNAs targeting CDK12 or CDK13. The qRT-PCR data in d and e were normalized to GAPDH. **f**, Flow cytometry analysis of γ-H2AX staining in IMR-32 NB cells treated with 400 nM THZ531 for the indicated time points (left). Gating was performed as shown in the left panel. Numbers indicate the percentages of living cells stained positive for γ-H2AX. Quantification of staining (right). Throughout the figure, error bars indicate mean values ±SD of two or three experiments, *$p<0.05$, **$p<0.01$, ***$p<0.001$, two-tailed Student's t-test.

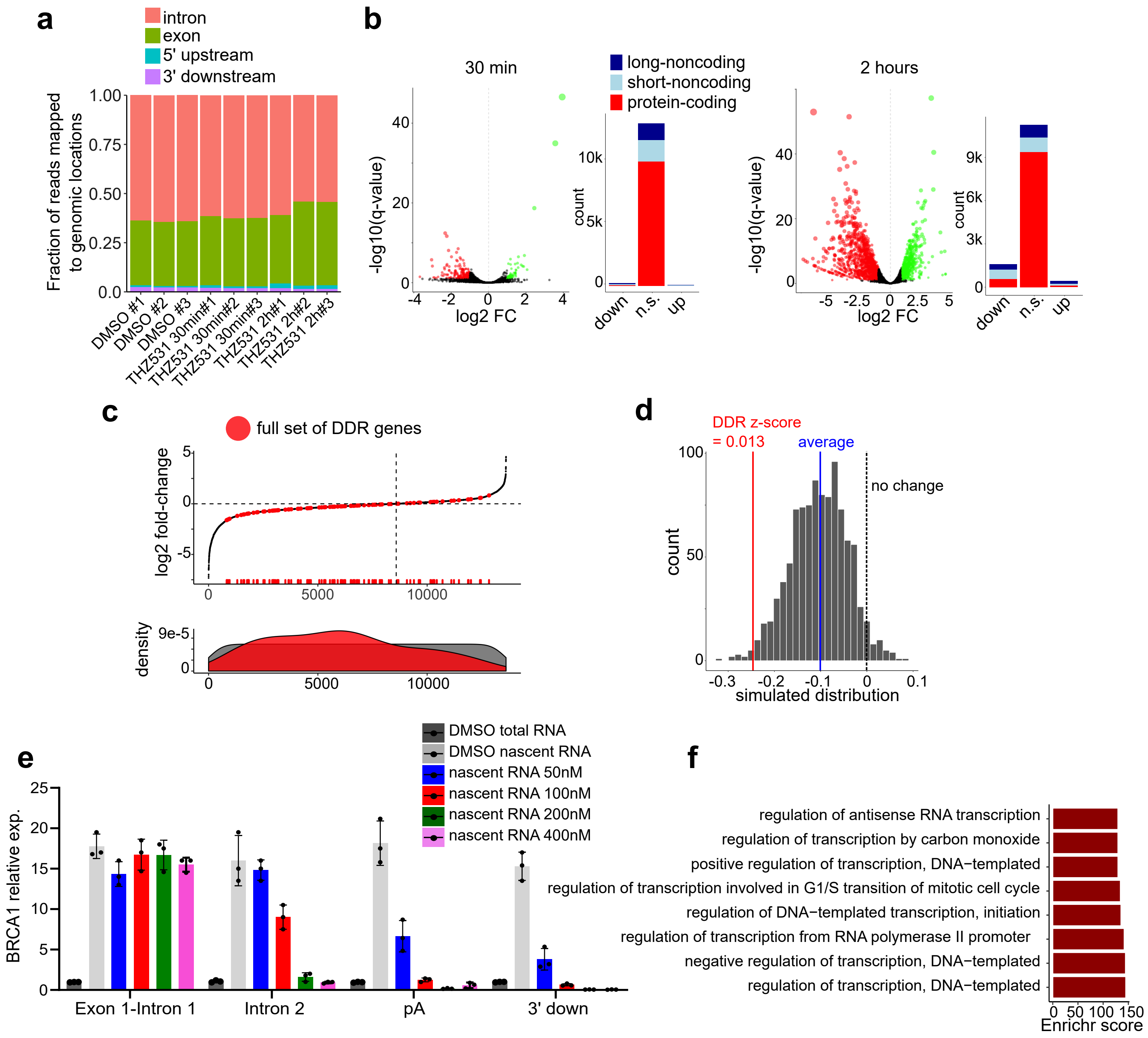

Supplementary Figure 3. **CDK12/13 inhibition results in genome-wide alterations in nascent RNA expression.** **a**, Bar plot showing the genomic distribution of TT-seq reads for each replicate of the three conditions tested (DMSO and THZ531, 400 nM for 30 min and 2 h). **b**, Volcano plot representation of differentially expressed genes following treatment with THZ531 for 30 min (left) and 2 h (right). The fold changes are represented in log2 scale (x-axis), and the −log10 q-value depicted on the y-axis (FDR < 0.1 and |log2 FC| >1). Bar plots depict the numbers of up- and downregulated genes for each aggregated gene group in cells treated with THZ531 for 30 min and 2 h. **c**, Waterfall (upper) and density (lower) plots of log2 fold-changes in gene expression in IMR-32 NB cells treated with 400 nM THZ531 for 2 h; the aggregated set of DDR genes is highlighted in red. **d**, Distribution of average simulated changes in gene groups of similar size to the aggregated set of DDR genes. The z-score for the DDR gene set is depicted in red. **e**, qRT-PCR analysis of nascent RNA expression at selected regions of the BRCA1 gene in NB cells treated with THZ531 at the indicated concentrations for 6 h. Error bars indicate mean values of ±SD, n=3. **f**, GO enrichment analysis of the top 400 downregulated genes after THZ531 (400 nM) treatment for 2 h.

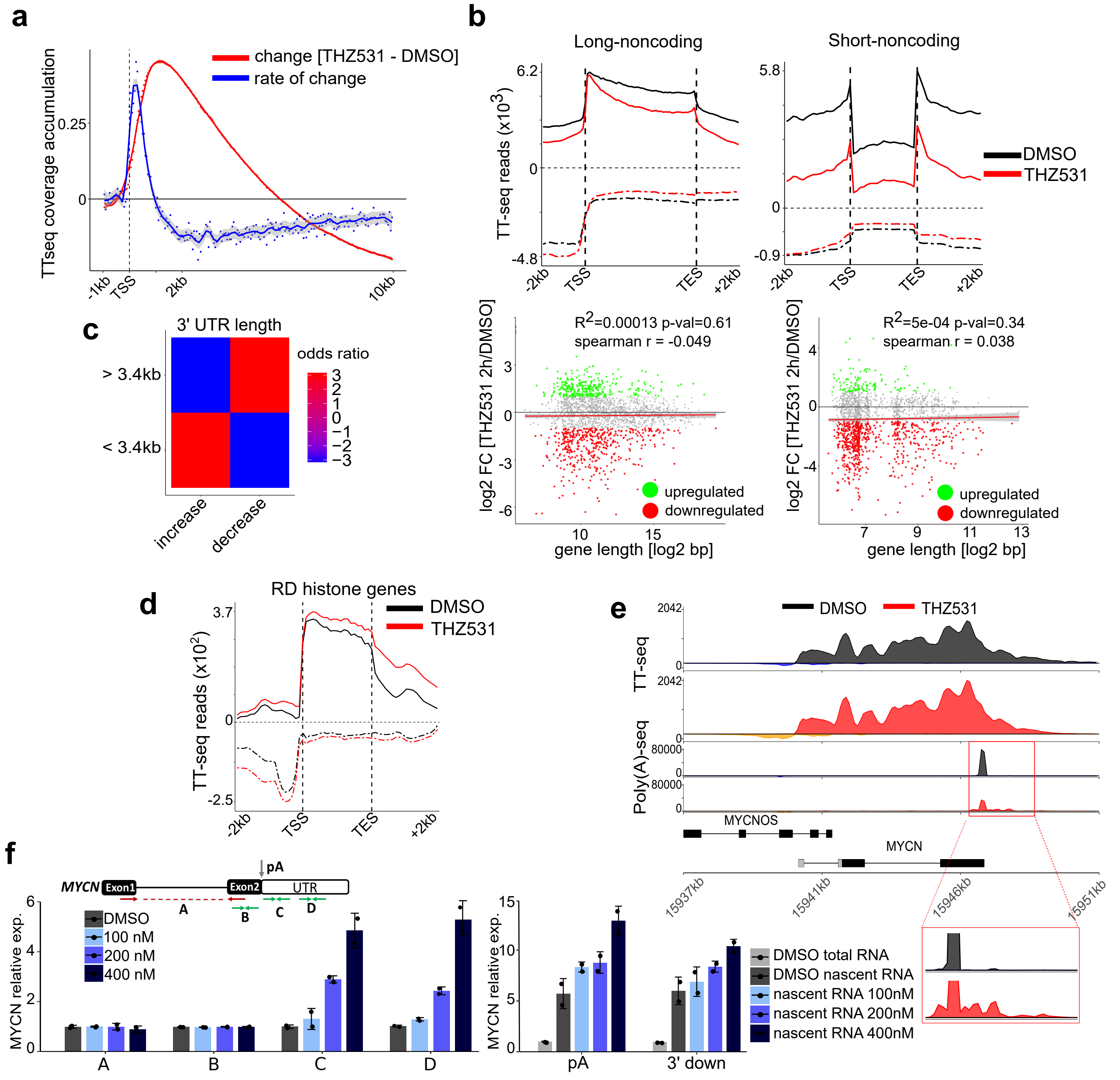

Supplementary Figure 4. **Noncoding and short genes do not exhibit a length-dependent elongation defect. a**, Average metagene profile depicting the change (red) and rate of change (blue) in TT-seq read densities in regions flanking the TSS (-1 kb to +10 kb) in cells treated with THZ531 (400 nM) for 2 h. **b**, Average metagene profiles of normalized TT-seq reads of long and short noncoding genes (top) in cells treated with THZ531 (400 nM) for 2 h. Associated scatterplots of log2 fold changes in gene expression versus gene length in log2 scales for each gene (bottom). Sense and antisense reads are depicted by solid and dashed lines, respectively. **c**, Heat map depicting the association between short genes and increased 3′ UTR length. Blue and red colors represent negative and positive odds ratios, respectively (Fisher's exact test). **d**, Average metagene profiles of normalized TT-seq reads of RD histone genes. **e**, TT-seq and poly(A) 3′-seq tracks at the MYCN locus showing 3′ UTR extension with usage of distal 3′ poly(A) sites upon treatment with THZ531 for 2 h. Enlarged box shows multiple poly(A) 3′-seq peaks downstream of the last dominant peak. **f,** qRT-PCR analysis of total RNA (left) and nascent RNA expression (right) at the indicated regions (A, B, C, D) of the MYCN gene in NB cells treated with THZ531 at the indicated concentrations for 6 h. Error bars indicate mean values ±SD, n=2.

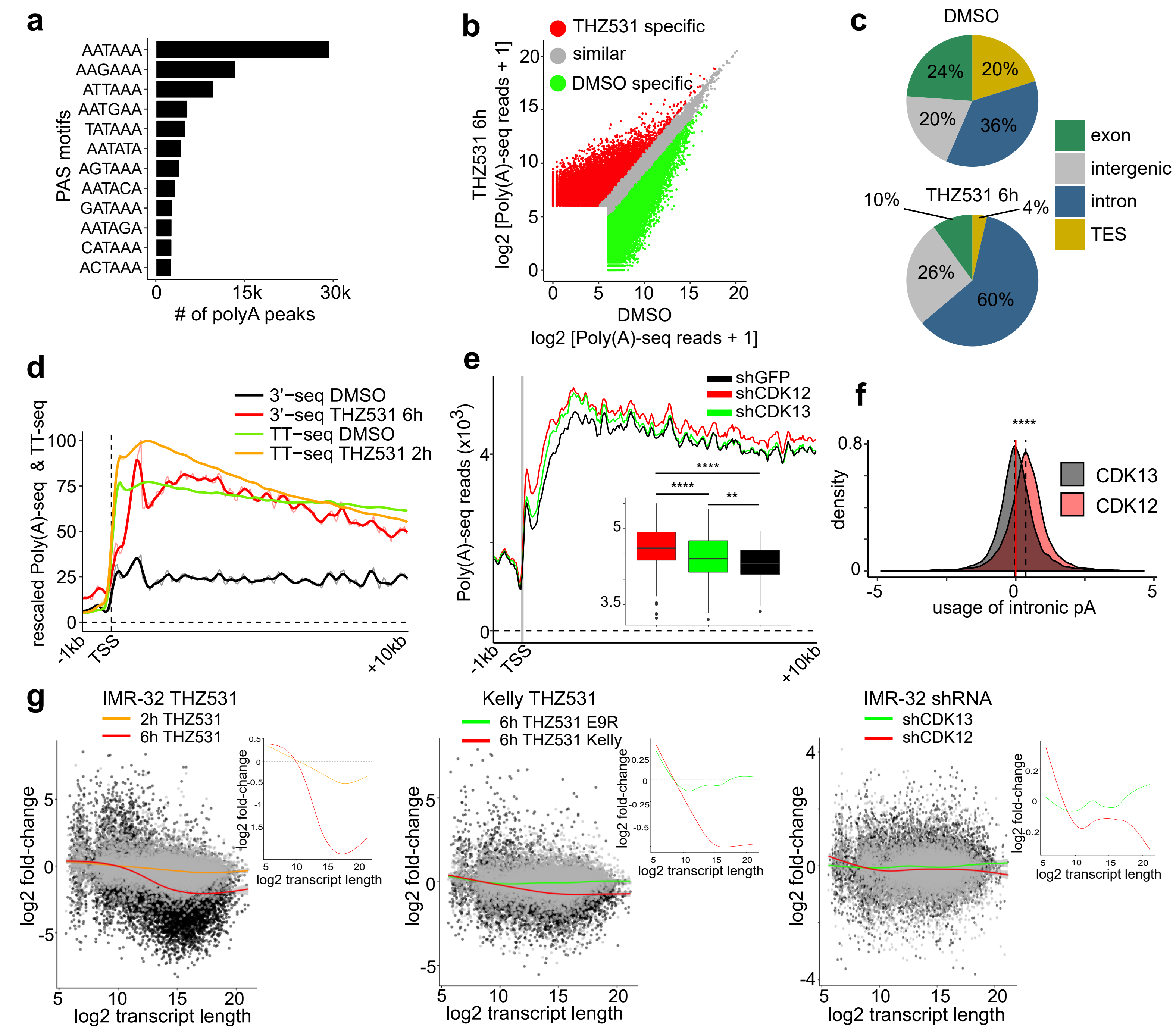

Supplementary Figure 5. **CDK12 depletion induces a higher usage of intronic polyadenylation sites. a**, Bar plots depicting the frequency of retrieved polyadenylation site (PAS) motifs 100 bp upstream of the poly(A) 3′-seq peaks. **b**, Scatterplot of poly(A) 3'-seq reads for all detected peaks in DMSO (x-axis) and THZ531-treated (y-axis) cells. Peaks with a 2-fold increase or decrease were considered to be THZ531- or DMSO-specific respectively. **c**, Pie charts depicting the genomic distributions of poly(A) 3'-seq peaks from panel b, in DMSO- and THZ531-treated cells. **d**, Average metagene profiles of rescaled (1-100) and normalized TT-seq and poly(A) 3′-seq reads at the TSS (- 1 kb to + 10 kb) for all PCPA genes in cells treated with DMSO or THZ531. **e**, Average metagene profiles of normalized poly(A) 3′-seq reads at the TSS (- 1 kb to + 10 kb) in IMR-32 NB cells expressing shRNAs against CDK12 and CDK13. Cells expressing a shRNA against GFP were used as controls. Inset, box plots depicting the change in distribution values over above-mentioned range (-1 kb to +10 kb) (shCDK12 vs. shGFP, ****p = 5.3e-13; shCDK13 vs. shGFP, **p = 0.01; shCDK12 vs. shCDK13, ****p = 4.8e-6; Wilcoxon rank-sum test). **f**, Density plots of odds ratios of poly(A) site usage (intronic vs 3′UTR) in the cells described in panel e (shCDK12 vs. shCDK13, p = 0; Kolmogorov-Smirnov test). **g**, Scatterplot of the log2 fold changes (vs. control) in gene expression versus gene length in log2 scale for each protein-coding gene in IMR-32 and Kelly NB cells under the following conditions: IMR-32 cells treated with THZ531, 400 nM (left), Kelly WT and Kelly E9R cells treated with THZ531, 200 nM (middle), for the indicated times; IMR-32 cells expressing shRNAs against CDK12 and CDK13 (right). For each condition, a generalized additive model (GAM) smoothing curve depicting the general trend in expression change is shown next to each scatterplot.

a

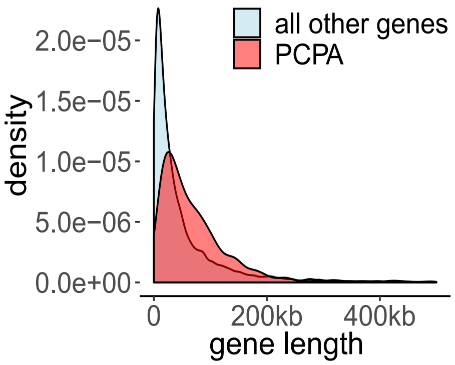

b

DNA Damage Response genes within the 809 genes that underwent PCPA following THZ531 treatment								
short	medium-short	medium-long			long			
NABP1	ERCC4	GEN1	POLH	CLSPN	FAM20C	EP300	VRK1	BRIP1
AP5S1	DYRK3	RBBP6	RAD51C	TBK1	PRKAA2	WAC	MARK1	REV3L
APTX	SLF2	DBF4	TOP3A	USP45	DTL	INTS7	INO80	NIPBL
	CDC6	SIRT1	MAML1	TICRR	CREB1	SMC5	FNIP2	PDS5B
	UPF1	ALKBH1	GTF2H1	LATS1	ERN1	ATF2	WRN	BCL2
	ATRIP	RBBP5	RAD17	NEK4	USP10	BLM	MMS22L	BRAF
	RMI1	STK17B	HELB	ALKBH8	ORC5	NUP98	COL4A3BP	MSH3
	DDB2	PALB2	KAT7	TOPBP1	BAZ1B	BAZ1A	MAP3K20	PRKCA
	ING3	FAN1	EXO1		BARD1	EP400	NLK	
		ALKBH3	MCM8		BRCA2	HUNK	MAPK4	

c

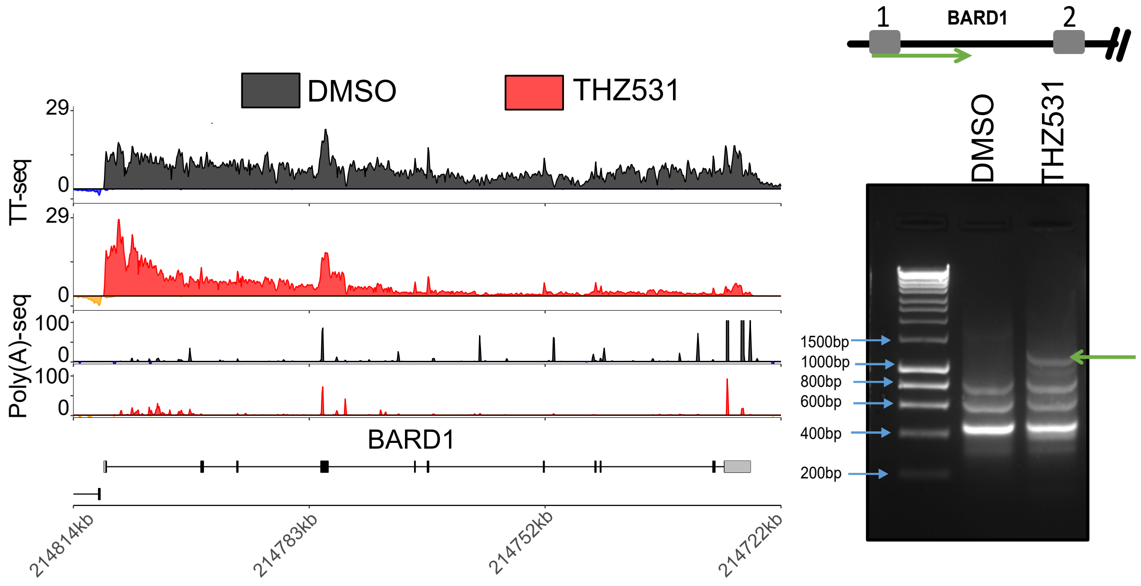

Supplementary Figure 6. CDK12 depletion results in a gene length-dependent loss of expression. a, Density plot showing the distribution of gene length among all genes vs. that among PCPA genes ($p = 2e\text{-}11$, Wilcoxon rank-sum test). **b**, Table depicting the identified DDR genes within the PCPA gene group and stratified according to the gene length groups established in Fig. 2d. **c**, TT-seq and poly(A) 3'-seq tracks at the BARD1 locus showing loss of annotated terminal polyadenylation signal and early termination due to PCPA upon treatment with THZ531 (400 nM, 6 h) (left). Agarose gel electrophoresis of 3'-RACE products of BARD1 in NB cells treated with THZ531 or DMSO (right). PCR products were validated by DNA sequencing. Arrow indicates the isoform with intronic poly(A) site.

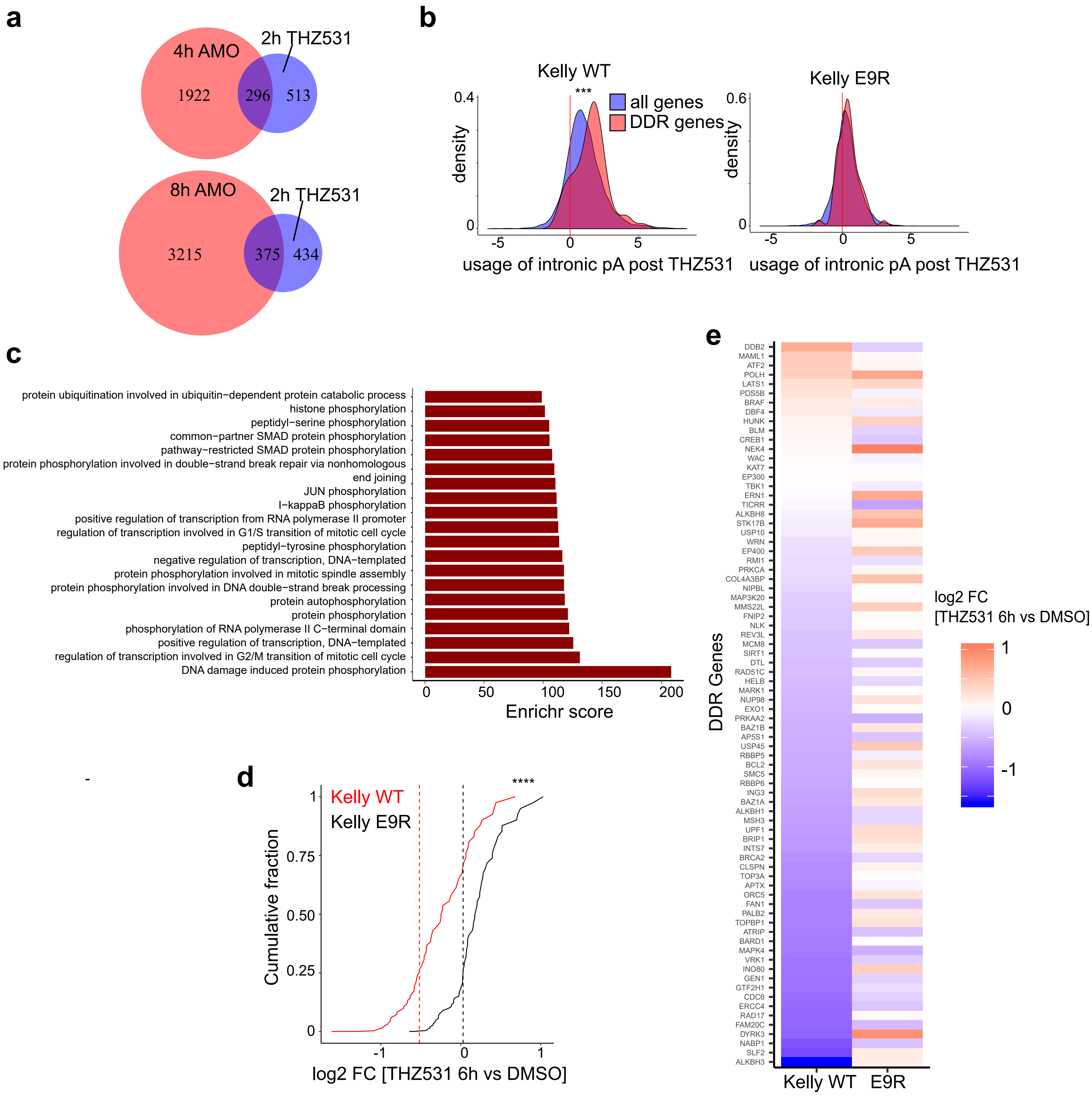

Supplementary Figure 7. CDK12 regulates the processing of DDR gene transcripts. a, Comparison of PCPA genes identified in Oh et al.[1] after U1 inhibition with a U1 antisense morpholino oligonucleotide (AMO) at 4 h ($p = 7.52e{-52}$ and odds ratio = 3.5) and 8 h ($p = 2e{-38}$ & odds ratio = 2.7) with PCPA genes identified in TT-seq analysis of cells treated with THZ531 400 nM for 2 h (all comparisons by Fisher's exact test). **b**, Density plots of odds ratios of poly(A) site usage (intronic vs 3'UTR) in all genes (blue) vs. DDR genes only (red) in Kelly WT and Kelly E9R cells treated with THZ531 200 nM for 6 h ($p = 5.6e{-4}$ and $p = 0.4$, respectively; Kolmogorov-Smirnov test). **c**, GO enrichment of genes that display increased intronic poly(A) usage in Kelly WT cells described in panel b. (FDR < 0.01). **d**, Cumulative fraction plots illustrating changes in the expression of DDR genes in Kelly WT and Kelly E9R cells (****$p = 3.3e{-16}$, Kolmogorov-Smirnov test). **e**, Heat map of log2 fold changes in gene expression values in Kelly WT and Kelly E9R cells treated with THZ531 (400 nM, 6 h) vs. DMSO.

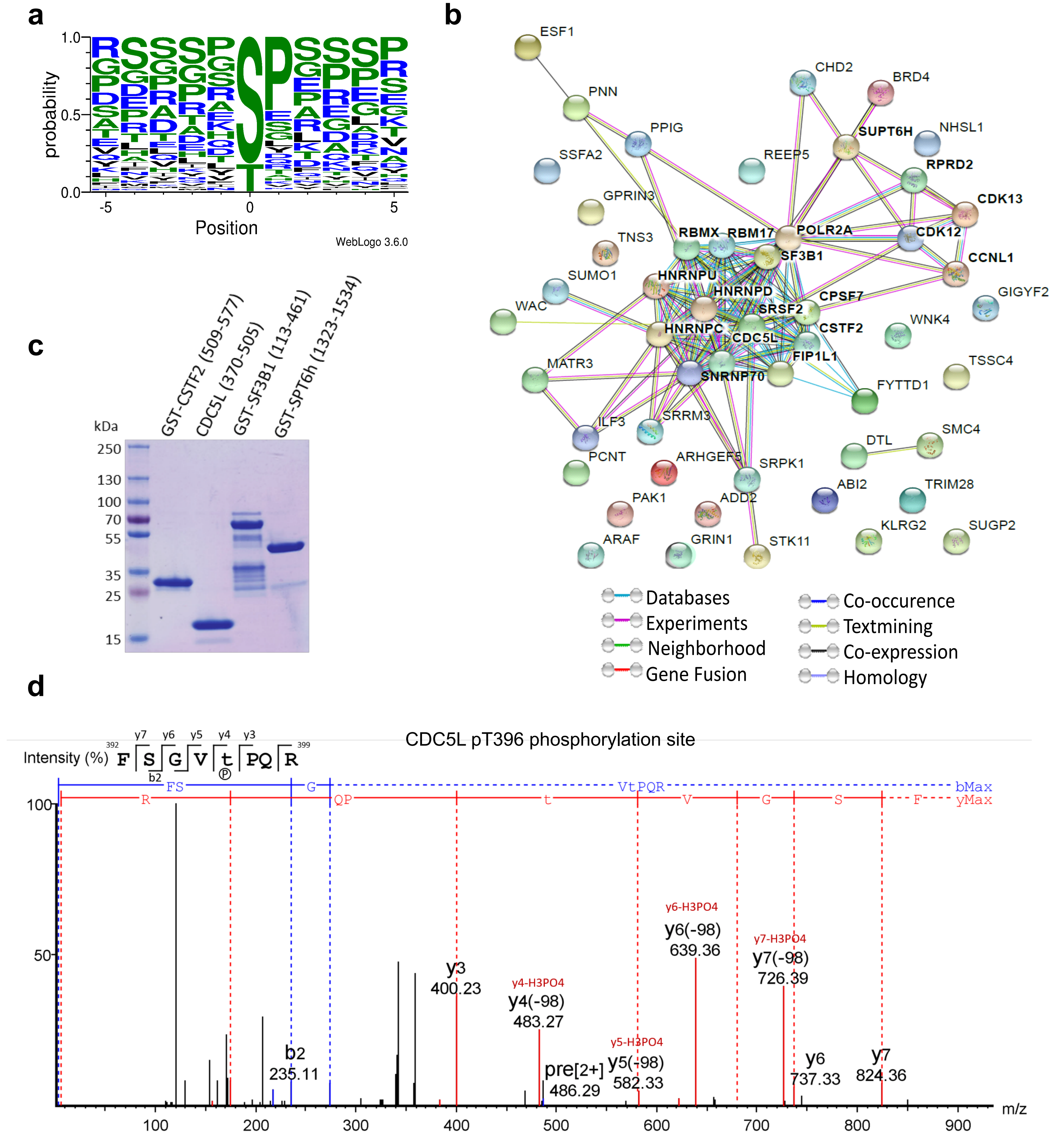

Supplementary Figure 8. **CDK12/13 inhibition affects phosphorylation of RNA processing genes. a**, Consensus sequences derived from the phosphopeptides showing decreased phosphorylation in cells treated with THZ531 (400nM, 2 h) using WebLogo 3 software, (http://weblogo.threeplusone.com/create.cgi). The phosphorylation site detected in SILAC analysis is labeled as position 0 in the plot. **b**, Protein-protein interaction network of the candidate phosphorylation substrates identified from SILAC analysis of NB cells as in panel a, using the STRING 10.5 database (http://string-db.org/). **c**, Coomassie blue staining of GST-tagged recombinant proteins used in the in vitro kinase assays. SPT6H was initially identified as a potential substrate of CDK12/13, but was not confirmed in subsequent studies. **d**, Mass spectrometry spectra generated using Xcalibur software (Thermo Fisher Scientific) of a specific CDC5L peptide incubated with CDK12/CycK. The phosphorylation site (pT396) identified by mass spectrometry is indicated.

Supplementary Figure 9 part 1

Figure 1b

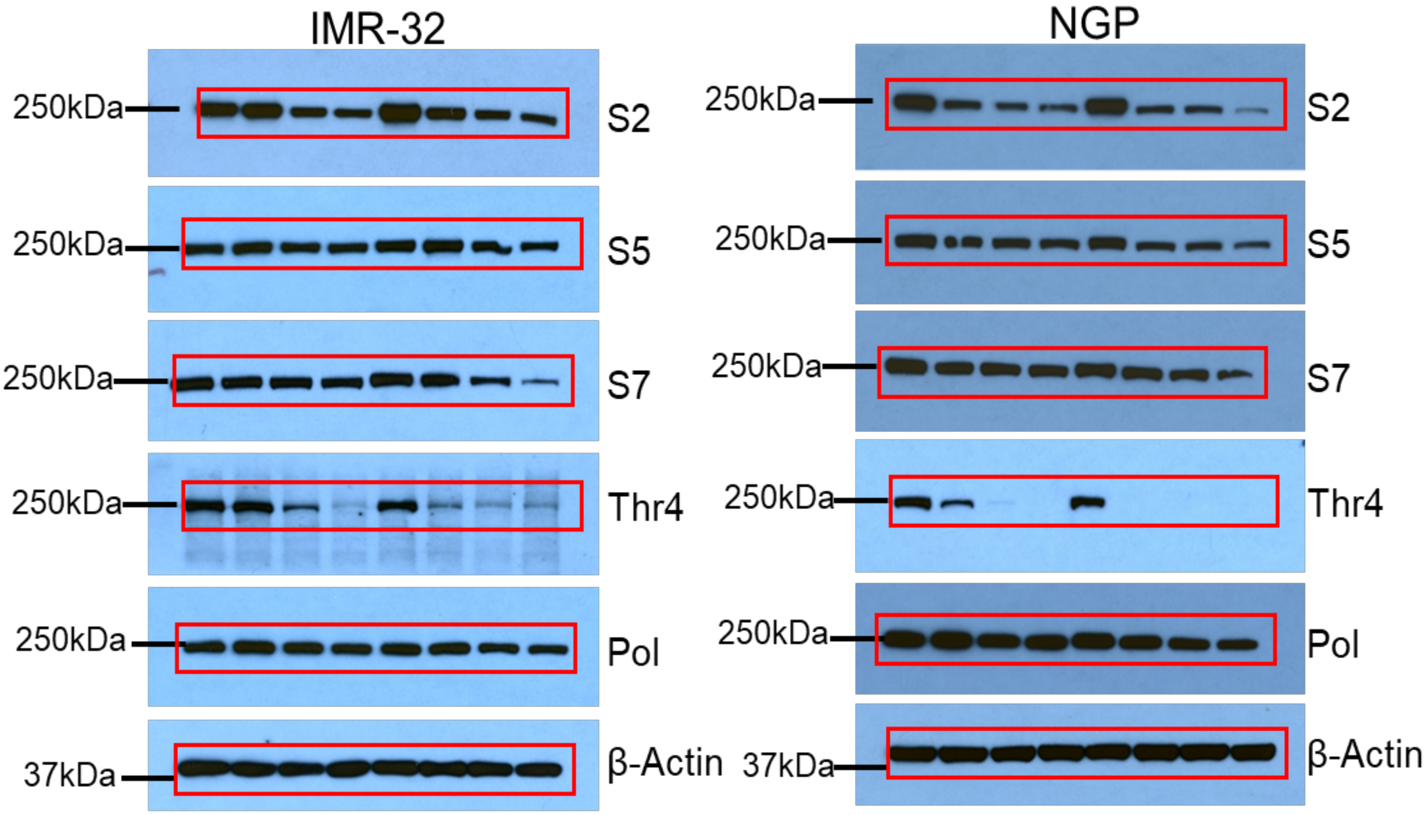

Supplementary Fig. 1b

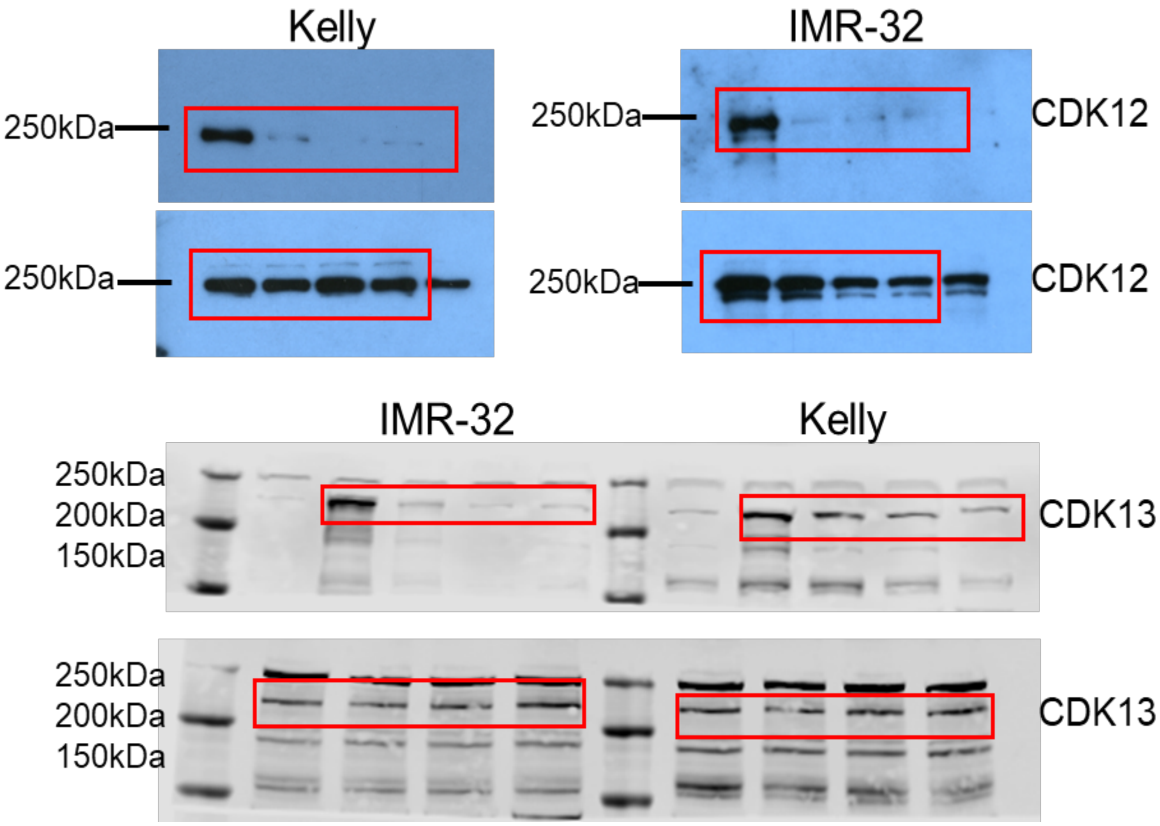

Supplementary Figure 9 part 2

Supplementary Fig. 1d

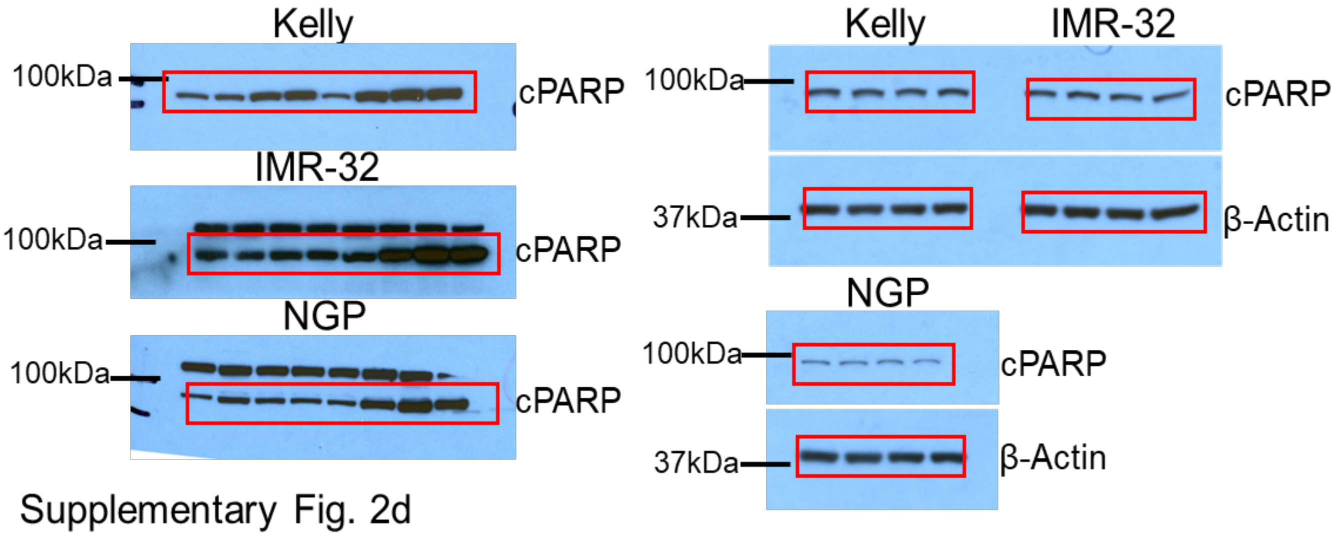

Supplementary Fig. 2d

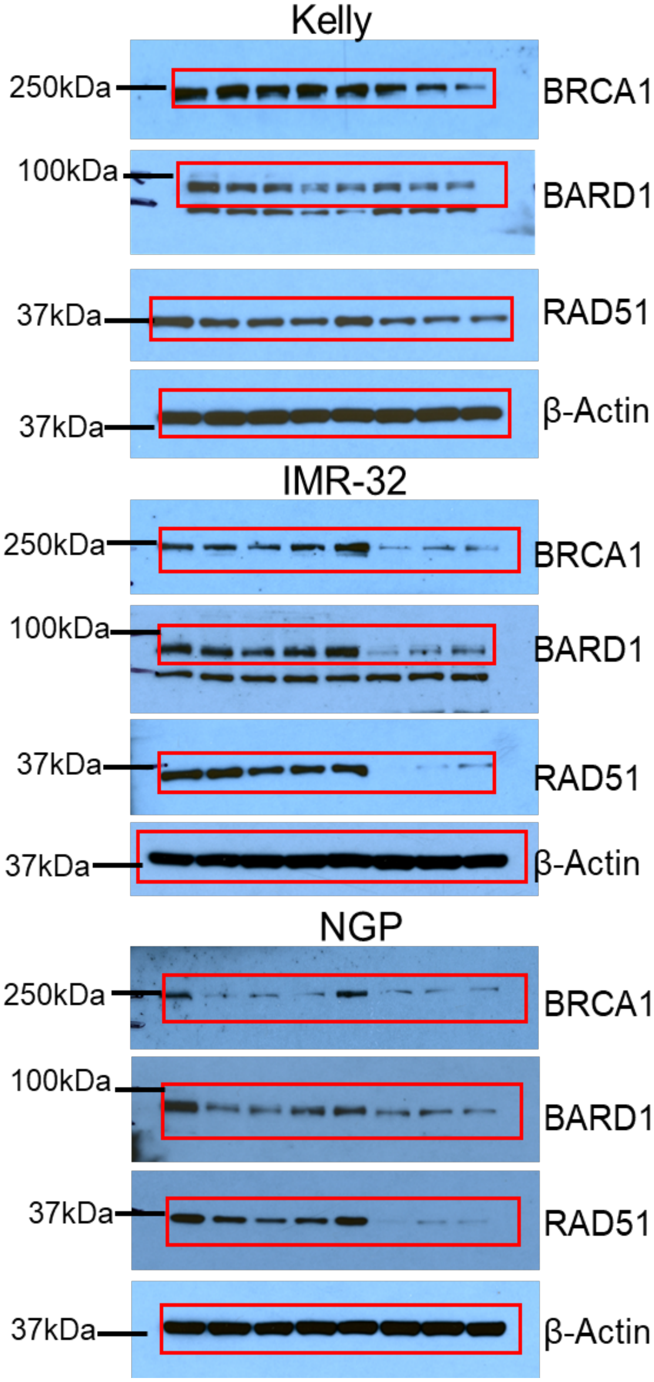

Supplementary Fig. 2e

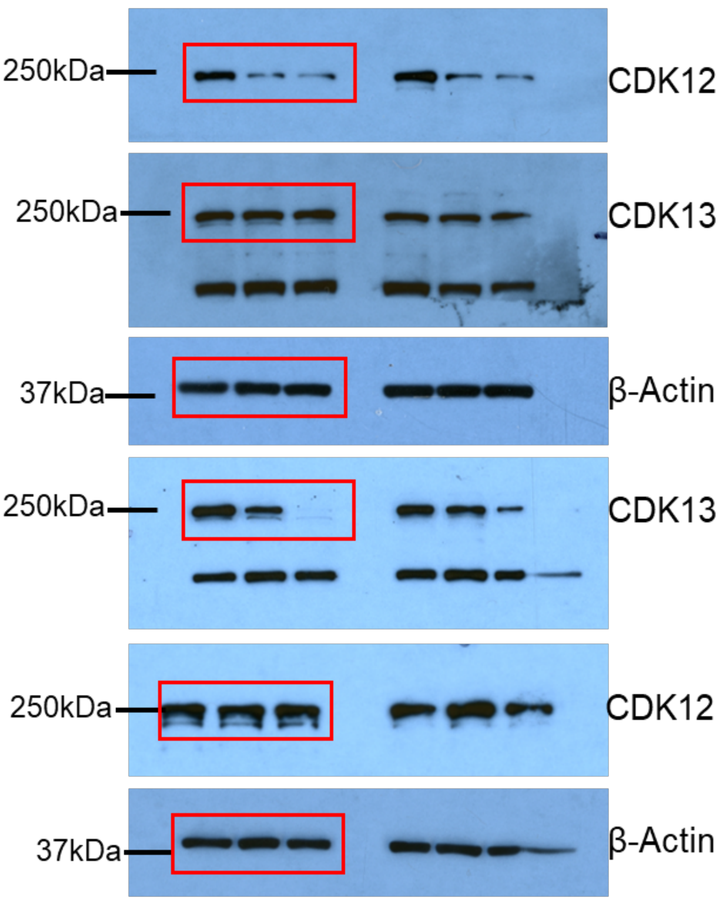

Supplementary References

1. Oh, J. M. et al. U1 snRNP telescripting regulates a size-function-stratified human genome. Nat Struct Mol Biol 24, 993-999, doi:10.1038/nsmb.3473 (2017).
