# Supplementary material for: CDK12 loss in cancer cells affects DNA damage response genes through premature cleavage and polyadenylation
Source: Nat Commun. 2019 Apr 15;10:1757. doi: 10.1038/s41467-019-09703-y (PMC6465371; doi:10.1038/s41467-019-09703-y)
Supplement: Supplementary file 3 — Reporting Summary [file 41467_2019_9703_MOESM3_ESM.pdf]

nature research

Corresponding author(s): Rani George
Last updated by author(s): 2019/02/26

Reporting Summary

Nature Research wishes to improve the reproducibility of the work that we publish. This form provides structure for consistency and transparency in reporting. For further information on Nature Research policies, see Authors & Referees and the Editorial Policy Checklist.

Statistics

For all statistical analyses, confirm that the following items are present in the figure legend, table legend, main text, or Methods section.

n/a	Confirmed	
☐	☒	The exact sample size (n) for each experimental group/condition, given as a discrete number and unit of measurement
☐	☒	A statement on whether measurements were taken from distinct samples or whether the same sample was measured repeatedly
☐	☒	The statistical test(s) used AND whether they are one- or two-sided<br>*Only common tests should be described solely by name; describe more complex techniques in the Methods section.*
☒	☐	A description of all covariates tested
☐	☒	A description of any assumptions or corrections, such as tests of normality and adjustment for multiple comparisons
☐	☒	A full description of the statistical parameters including central tendency (e.g. means) or other basic estimates (e.g. regression coefficient) AND variation (e.g. standard deviation) or associated estimates of uncertainty (e.g. confidence intervals)
☐	☒	For null hypothesis testing, the test statistic (e.g. F, t, r) with confidence intervals, effect sizes, degrees of freedom and P value noted<br>*Give P values as exact values whenever suitable.*
☒	☐	For Bayesian analysis, information on the choice of priors and Markov chain Monte Carlo settings
☒	☐	For hierarchical and complex designs, identification of the appropriate level for tests and full reporting of outcomes
☐	☒	Estimates of effect sizes (e.g. Cohen's d, Pearson's r), indicating how they were calculated

Our web collection on statistics for biologists contains articles on many of the points above.

Software and code

Policy information about availability of computer code

Data collection	For analysis in Supplemental Figure 7a, a list of PCPAed genes were retrieved from Oh, J. M. et al. (Nat Struct Mol Biol. 2017)
Data analysis	STAR (v2.5.1b_modified), samtools (v1.3.1), DeepTools (v2.5.4), MACS (v2.1.1), Kallisto (v0.43.1), FeatureCounts (v1.5.0-p1), rMATS (v4.0.1); R packages: DESeq2, data.table, GeTrackViz2, GenomicRanges, Biostrings

For manuscripts utilizing custom algorithms or software that are central to the research but not yet described in published literature, software must be made available to editors/reviewers. We strongly encourage code deposition in a community repository (e.g. GitHub). See the Nature Research guidelines for submitting code & software for further information.

Data

Policy information about availability of data

All manuscripts must include a data availability statement. This statement should provide the following information, where applicable:

- Accession codes, unique identifiers, or web links for publicly available datasets
- A list of figures that have associated raw data
- A description of any restrictions on data availability

Microarray, TT-seq, Poly(A) 3′-seq datasets have been deposited in the Gene Expression Omnibus (GEO), accession number GSE113314. The SILAC dataset has been deposited in the ProteomeXchange Consortium, accession number PXD009533. All other data are available from the corresponding author upon request.

Field-specific reporting

Please select the one below that is the best fit for your research. If you are not sure, read the appropriate sections before making your selection.

☒ Life sciences ☐ Behavioural & social sciences ☐ Ecological, evolutionary & environmental sciences

For a reference copy of the document with all sections, see nature.com/documents/nr-reporting-summary-flat.pdf

Life sciences study design

All studies must disclose on these points even when the disclosure is negative.

Sample size	For all experiments, sample size pre-calculation was not performed. These numbers are sufficient to obtain statistical differences or effect sizes.
Data exclusions	No data were excluded from the analyses.
Replication	To compensate for experimental variation, most experiments were performed at least three times including technical replicates. There are no findings that were not replicated
Randomization	For our experiments, no randomization was applied.
Blinding	Investigators were not blinded to group allocation or data analysis.

Reporting for specific materials, systems and methods

We require information from authors about some types of materials, experimental systems and methods used in many studies. Here, indicate whether each material, system or method listed is relevant to your study. If you are not sure if a list item applies to your research, read the appropriate section before selecting a response.

Materials & experimental systems

n/a	Involved in the study
☐	☒ Antibodies
☐	☒ Eukaryotic cell lines
☒	☐ Palaeontology
☒	☐ Animals and other organisms
☒	☐ Human research participants
☒	☐ Clinical data

Methods

n/a	Involved in the study
☒	☐ ChIP-seq
☐	☒ Flow cytometry
☒	☐ MRI-based neuroimaging

Antibodies

Antibodies used	RNAPII CTD S2 (Bethyl cat# A300-654A, 1:10,000); RNAPII CTD S5 (Bethyl cat# A300-655A, 1:10,000); RNAPII (Santa Cruz cat# sc-899, 1:1000); RNAPII CTD S7 (Millipore cat# 041570, 1:10,000) RNAPII CTD Thr4 (Active Motif cat# 61361, 1:1000) cleaved PARP (Cell Signaling cat# 9541, 1:1000); GAPDH (Cell Signaling cat# 2118S, 1:4000); CDK12 (Cell Signaling cat# 11973S, 1:1000); CDK13 (Bethyl cat# A301-458A, 1:1000); CDK13 antibody (1:1000) used for WB in Supplementary Fig. 1e was kindly provided by Dr. Arno Greenleaf; γ-H2AX (Cell Signaling cat# 9718, 1:200), RAD51 (GeneText cat# GTX70230, 1:1000), BRCA1 (Cell signaling cat# 9010S, 1:500), BARD1 (Santa Cruz Biotechnology cat# sc11438, 1:500), Alexa-488 (Molecular Probes cat#A11008, 1:300),
Validation	For all antibodies used, validation statements and relevant citations can be found on the manufacturere's website. Further validation of selected antibodies is supported by western blotting of knock-down experiments in the manuscript

Eukaryotic cell lines

Policy information about cell lines

Cell line source(s)	Cell Culture. Human neuroblastoma (NB) cells (Kelly, IMR-32, IMR-5, LAN-1, LAN-5, NGP, SK-N-AS, SH-SY5Y, SKNFI, CHLA-20, CHLA-15) were obtained from the Children's Oncology Group cell line bank. Human lung (IMR-90) and skin fibroblasts (BJ) were kindly provided by Dr. Richard Gregory (Boston Children's Hospital). NIH3T3 cells were purchased from the American Type Culture Collection (ATCC).
Authentication	The cell lines were authenticated through STR analyses.
Mycoplasma contamination	All cell lines were routinely tested for mycoplasma.

Commonly misidentified lines
(See ICLAC register)

No commonly misidentified cell lines were used

Flow Cytometry

Plots

Confirm that:

☒ The axis labels state the marker and fluorochrome used (e.g. CD4-FITC).

☒ The axis scales are clearly visible. Include numbers along axes only for bottom left plot of group (a 'group' is an analysis of identical markers).

☒ All plots are contour plots with outliers or pseudocolor plots.

☒ A numerical value for number of cells or percentage (with statistics) is provided.

Methodology

Sample preparation	For cell cycle and DNA damage analysis, cells were treated as indicted. After 2, 6 and 24h, cells were trypsinized and fixed in ice-cold 70% ethanol overnight at -20ºC. After washing with ice-cold phosphate-buffered saline (PBS), cells were incubated in PBS-0.5% Tween-20 with γ-H2AX antibody overnight at 4°C. Cells were subsequently washed and incubated with Alexa-488-conjugated secondary antibody for 45 min and then treated with 0.5 mg/ml RNAse A (Sigma-Aldrich) in combination with 50 µg/ml propidium iodide (PI, BD Biosciences). For apoptosis analysis cells were harvested and stained with PI and FITC-Annexin V according to the manufacturer's protocol (BD Biosciences).
Instrument	FACS Calibur (Becton Dickinson)
Software	Samples on the FACS Calibur were acquired using CellQuest sofyware. All samples were analyzed using FlowJo.
Cell population abundance	For all experiments, at least 50,000 cells were measured to obtain relevant numbers after gating.
Gating strategy	Live cells were initially gated on the FSC/SSC plots, then doublets are excluded in a FL2A-FL2W plot.

☒ Tick this box to confirm that a figure exemplifying the gating strategy is provided in the Supplementary Information.
