# Supplementary material for: CDK12 loss in cancer cells affects DNA damage response genes through premature cleavage and polyadenylation
Source: Nat Commun. 2019 Apr 15;10:1757. doi: 10.1038/s41467-019-09703-y (PMC6465371; doi:10.1038/s41467-019-09703-y)
Supplement: Supplementary file 4 — Description of Additional Supplementary Files [file 41467_2019_9703_MOESM4_ESM.pdf]

Description of Additional Supplementary Files

File Name: Supplementary Data 1
Description: Fold-change gene expression changes in Kelly and IMR-32 cells treated with THZ531 (400nM, 6h) versus control.

File Name: Supplementary Data 2
Description: TT-seq gene expression changes in IMR-32 cells treated with THZ531 (400nM, 2h) versus control.

File Name: Supplementary Data 3
Description: A list of DDR genes from publicly available databases.

File Name: Supplementary Data 4
Description: Comparison of PCPA genes identified in Oh et al. (Nat Struct Mol Biol, 2017) after U1 inhibition with a U1 antisense morpholino oligonucleotide (AMO) with PCPA genes identified in this study, in TT-seq analysis of cells treated with THZ531 (400 nM, 2 h).

File Name: Supplementary Data 5
Description: Log2 ratios of identified phosphopeptides by SILAC analysis in Kelly and IMR-32 cells treated with THZ531 (400 nM, 2h) versus control.

File Name: Supplementary Data 6
Description: Mass fingerprinting analysis of selected recombinant protein substrates.
